# The Influence of Technology on Mental Well-Being of STEM Teachers at University Level: COVID-19 as a Stressor

**DOI:** 10.3390/ijerph18189605

**Published:** 2021-09-12

**Authors:** Johanna Andrea Navarro-Espinosa, Manuel Vaquero-Abellán, Alberto-Jesús Perea-Moreno, Gerardo Pedrós-Pérez, Pilar Aparicio-Martínez, Maria Pilar Martínez-Jiménez

**Affiliations:** 1 Unidad de Seguimiento a Graduados, Banca Laboral y Prácticas Pre Profesionales, Universidad de ECOTEC, Guayaquil 090501, Ecuador; jnavarro@ecotec.edu.ec; 2 GC12 Clinical and Epidemiological Research in Primary Care, Instituto Maimónides, Campus de Menéndez Pidal, Universidad de Córdoba, 14071 Córdoba, Spain; 3 Departamento de Enfermería, Fisioterapia y Farmacología, Campus de Menéndez Pidal, Universidad de Córdoba, 14071 Córdoba, Spain; 4 Departamento de Física Aplicada, Radiología y Medicina Física, Edificio Albert Einstein, Campus de Rabanales, Universidad de Córdoba, 14071 Córdoba, Spain; aperea@uco.es (A.-J.P.-M.); fa1pepeg@uco.es (G.P.-P.); fa1majip@uco.es (M.P.M.-J.); 5 Responsable Grupo Investigación PAIDI de la Junta de Andalucía TEP149, Modelos de Simulación en Energías, Transporte, Física, Ingeniería y Riesgos Laborales, Edificio Albert Einstein, Campus de Rabanales, Universidad de Córdoba, 14071 Córdoba, Spain

**Keywords:** mental health, mixed-method, STEM teachers, university, COVID-19

## Abstract

Stress can result in psychopathologies, such as anxiety or depression, when this risk factor continues in time. One major stressor was the COVID-19 pandemic, which triggered considerable emotional distress and mental health issues among different workers, including teachers, with another stressor: technology and online education. A mixed-method approach is presented in this research, combining a cross-sectional study of university teachers from Ecuador and Spain with a medium of twenty years of working experience (*N* = 55) and a bibliometric analysis carried out in three databases (161 documents). The levels of anxiety and depression, and therefore the risk of developing them as mental disorders, were high. The lack of training (*p* < 0.01), time (*p* < 0.05), or research regarding the use of technology in education (*p* < 0.01) and stress caused by COVID-19 (*p* < 0.001) were linked to frequency. The most relevant observational study obtained through the bibliometric analysis (138 citations and over 65% of methodological quality) indicated that previous training and behavioral factors are key in the stress related to technology. The combination of the results indicated that mental health in STEM teachers at university is related to diverse factors, from training to the family and working balance.

## 1. Introduction

The occupational safety and health (OSH) standards indicated the obligation of any employer to maintain both the physical and psychological well-being of the employees in the working environment [1,2]. Despite the guidelines and the legal framework, the adaptation of the working environment, especially to maintain mental health, continues to be limited or inadequate [3,4]. The problem resides in the fact that psychological and psychiatric disorders of workers impact their physiological and biological health as well as the productivity of the corporate, company, or employer [5,6]. In this sense, different authors have indicated how the prevention or treatment of mental disorders and their psychological component [7], including the adequation of the working place, equally contributes to workers’ well-being [8,9].

One significant aspect that favors alterations of the mental health area is stress [5]. Stress, a physiological response to internal or external factors or stressors, provokes the triggering of the sympathetic system, causing the reaction of the autonomous nervous system and the hypothalamic-pituitary-adrenal (HPA) gland axis [10,11]. This stimulation results in neurologic, endocrinologic, psychological, cognitive, and behavioral reactions, from increase of blood pressure, heart rate, blood glucose, vigilance or alertness, to containment of inflammatory or immune response [12,13]. The concerning issue is when this stimulation of the nervous system and HPA axis continues for medium and long periods, resulting in major health issues, such as infertility [14]. Although most stressors, both interior and exterior, tend to have short duration effect [15], the continuous stress exposition may be derivate in psychopathologies depending on the individual responses, therefore, causing anxiety [16], depression [17], and other mental health disorders [18].

One major exterior stressor was the COVID-19 pandemic [19,20,21], which triggered considerable emotional distress and mental health issues among different workers [22,23]. Most researchers have analyzed the impact of this pandemic, focusing on the healthcare workers [24,25,26], since they were the more exposed and had a higher risk of accidents. Despite the mental impact that occurred in the hospitals and healthcare sector, other employees also suffered from emotional and mental distress caused by the lockdown and the requirements of the working. Among the numerous areas impacted by COVID-19 [27], education took a toll since the educational centers and teachers had to change from face-to-face education to online teaching [28,29,30], without the proper tools or training in most cases.

In this sense, several studies indicated how stress or anxiety levels were higher than expected among teachers in the different educational levels [17,31,32]. This prevalence was represented as emotional tiredness, fatigue, headaches, sleep problems, eating or appetite alterations or disorders, irritability, exasperation, depressing feelings, or inability to focus [17,31,32]. One factor contributing to these emotional and mental issues is the technology or the information and communication technologies (ICTs), an external stressor [33]. The technology as a stressor is called technostress, described as a significant factor contributing to stress even before the pandemic [34]. In this case, minor studies have been carried out to determine the effect of technostress [35,36]. Only a recent study has pointed out how technostress and the burnout syndrome are reduced when the teachers have higher levels of technological pedagogical knowledge, being explained as 70% of the current prevalence [37]. Therefore, the factor that seems to increase the levels of anxiety, stress, and depression [17,33] among teachers appears to be the technology [37]. One study indicated how teachers from science, technology, engineering, and mathematics (STEM) backgrounds, such as engineering degrees, had less risk since their ICTs’ skills or training protect them against the technostress [38]. A Turkish study (2020) indicated how technological knowledge, which is higher among STEM teachers, decreases the probability of developing emotional problems and technostress [39]. Despite these results and relevance regarding technology and mental health, researchers continue not studying the teachers’ perception regarding technology, such as frequency of use or skills, as factors that may contribute to the stress or decrease the effect of technostress [36,37].

Another factor contributing to emotional or mental health was the lack of resources or training, but few studies in higher education have indicated the teachers’ perspective regarding these difficulties [40,41]. The issue is whether these teachers’ mental risks could be managed through ICTs training and the availability of resources.

## 2. STEM Education, STEM Teachers, and Mental Health

Despite being present for the last 30 years and being highly discussed, STEM education has been recently conceptualized and defined [42]. One of the complete definitions is the one given by Moore et al. [43] that defines it as “the teaching and learning of the content and practices of disciplinary knowledge, which include science and/or mathematics through the integration of engineering and engineering design practices of relevant technologies”. This definition in combination with other authors [44,45] reflect how STEM education incorporates not only the teaching of scientific or mathematical concepts but also the engineering use and design of technology as well as the skills needed to adequately use such tools [42]. Despite this, Bybee [46] indicates that perhaps this definition depends on the individual’s context and needs, being modifiable, subject to the current moment.

Besides, a STEM teacher, which can be defined as an educator with a curriculum based on the four disciplines (science, technology, engineering, and mathematics) and understanding of the STEM education, should use technology and know the engineering design that provide the base for the ICTs [42]. Therefore, a STEM teacher is not only someone that instructs different scientific knowledge and skills, but rather has previous training and reasonable comprehension of the dynamics and bases of ICTs in education [47]. Usually, STEM teachers are more commonly framed in elementary or high school education, although the definition of this type of teacher is also applicable to the higher education institutions [48]. Nevertheless, there is no precise description for STEM teachers, much less a conceptualization of STEM teachers at university level [49].

Despite the lack of conceptualization of the figure, several studies have analyzed the perception and aptitudes of pre-service STEM teachers, mainly in the primary and secondary educational level [42,44,46,50] that indicated a need for training. Only one article [51] focused on in-service teachers, both STEM and non-STEM, which showed a concern about the engineering course within STEM disciplines since many STEM and non-STEM teachers had low levels of engineering bases, which contradicts the definition of STEM education [43]. Berisha and Vula [50] indicated in their study how pre-service teachers that received a course in this methodology perceived a lack of collaboration or unwillingness as challenges to implement this education. In this sense, teachers with a STEM background usually avoid including new educational methodologies, such as flip classrooms, gamification, or blended learning, since they tend to carry out the same educational methodologies that they were taught [51]. One reason for the reticence of teachers is that STEM teaching has, as a purpose, deep understanding of science and/or mathematics content, engagement of students through the educational pedagogics and ICTs, and improvement of communication skills and teamwork among students [52]. These teachers’ expectations could provoke high levels of stress and other psychological issues [51]. Despite this risk, most studies have indicated that STEM teachers tend to have fewer mental issues, possibly for the training received during the early stages of their career [53]. The previous data showed that STEM teachers in mainly primary and secondary educational levels had lower levels of anxiety and other emotional issues related to their ICTs’ knowledge, skills, and training [36,38,39]. These studies also focused on mixed approaches, including surveys and interviews, to further understand the relationship between these workers’ perception and their mental health, who seemed to be more prepared against the stress related to ICTs [37,38,39].

Nevertheless, these teachers’ skills, knowledge, or ability might not have had to be at the highest level when the pandemic struck the world [29,30,41]. The pandemic of COVID-19 obliged to switch the educational process from face-to-face to online, putting further pressure and stressor on the teachers at any educational level [17,38]. Several studies have analyzed psychological impact, such as stress or anxiety, among teachers from high schools, with relevance to Latin America or Spain [17,54,55]. Little studies have focused on the in-service teachers at the university level and their psychological status [18,56]. Other researchers have analyzed the university teachers’ skills or resources available at the beginning of the pandemic [57,58], including two intercontinental researches focusing on Ecuador and Spain whose primordial population were students [59,60]. In these two articles [59,60] carried out by the same authors, the results indicated how students and university teachers indicated that they had faced difficulties, being extremely significant to the lack of training or the lack of teachers’ digital skills. This comparison between the two countries is achievable since the educational systems, laws, and structure are similar [61], showing that the deficiency of resources or training for the switch to online education seemed to be present at the same percentage across countries [57,58].

Another aspect that has been highlighted in previous studies is the importance of reviewing the scientific content, especially in education during the pandemic [62]. This fact was already pointed out by Milner-Bolotin [63], who emphasized the relevance of review and accessible evidence regarding STEM teaching and the education or training that these employees had. Nonetheless, no study has reviewed neither analyzed any intercontinental differences, mainly Ecuador and Spain, regarding the emotional impact of in-service university teachers with high experience in STEM education (e.g., university teachers in engineering courses), regarding their perception about skills or absence of resources and being framed in the STEM education. In accordance with the absence of information and the significance of reviewing scientific content [63], the current research has implemented a mixed-method combining the study and recruiting of new data and bibliographic analysis of current research focusing on this topic in order to obtain a better comprehension of the results.

Therefore, and based on the lack of evidence regarding the teachers’ psychological impact of COVID-19 at the university level, the role of STEM and their training and no current analysis of previous studies, the current research had as objectives:

O1. To analyze the level of anxiety and depression among teachers with high expertise in STEM education, and therefore, high level of ICTs’ skills and engineering understanding.
O2. To determine the associations between the level of anxiety and depression, and their risk related to the difficulties that this population may perceive, such as the deficiency of training, resources or the stress caused by COVID-19.
O3. To review the current knowledge available about the ICTs, university, and their mental health and comprehend the results obtained in the observational study.
O4. To examine the importance of STEM education and its training as a protective factor to mental problems among university teachers.

## 3. Materials and Methods

### 3.1. Sample, Data Collection, and Survey

A transverse descriptive investigation was implemented through a target population of teachers with a median of twenty years of working experience in different institutions from Spain and Ecuador (Table 1). The selection of the sample and institutions was founded on the fact that teachers with a STEM background seemed to have more ICTs’ skills, and therefore, have lower risk of mental health issues related to the technostress [64,65]. The survey was distributed from September to October 2020 after the first lockdown in both countries. This non-probabilistic sampling had, as selection criteria, the accessibility and the relevance according to international ranking since the positions of institutions in this ranking would imply higher levels of ICTs’ skills and accessibility of resources. These criteria delimited to universities from Spain and Ecuador with an interest for Engineering courses, being selected two universities with higher rankings in STEM education (Madrid and Valencia), two in the middle and two in the lower section of the ranking (Cordoba and Ecuador) [66]. The invitation to participate was sent to different teachers from STEM faculties, mainly Engineering, since the level of ICTs’ skills and engineering knowledge should be the highest, and after receiving confirmation and willingness to participate, the survey link was sent. Additionally, these participants were asked to distribute the survey among other colleges working in the same school, providing a randomness to the sample. The sample of teachers that accessed the survey was 173, however, only 90 teachers started the survey (Table 1). Out of these 90 teachers, two indicated “No” to give their consent to participate and 20 only filled two questions. The remaining 70 teachers completed at least a minimum of 5 questions, with the questionnaire completely filled by 55 teachers, most of them from Ecuador. The teachers who accepted to contribute were from the top, medium, and bottom of the ranking; three were from Spain and one from Ecuador.

This study was completed through an original and specific online survey, which included informed consent and the study’s objective. The survey included 11 items focused on ICTs and education (ICTs’ skills, frequency of using ICTs, opinion regarding lack of ICTs), the impact of COVID-19 on their level of stress (“Do you consider that COVID-19 has increased your stress level?”), and the anxiety and risk of depression scales. The online survey was distributed via QuestionPro (Survey Analytics LLC, San Francisco, CA, USA). The first page of the survey was formed by the information about the research, its purpose, ethical code, confidentiality, and anonymization. After the agreement to partake in the research, the participants were redirected to the items of the questionnaire; it was possible to unmark or indicate ‘unknown’ for each question. The survey included teachers’ opinions about ICTs (frequency of using ICTs, relevance of the ICTs and role in education, ICTs’ skills, and obstacles of using ICTs), whose validity, reliability, and consistency was acceptable [69]; the next section included the perception of COVID-19 in stress levels (with a response of Yes, No or Maybe), a Linkert scale (from 1 = No stress to 5 = Maximum stress) of stress caused by the imposition of ICTs, the technical issues suffered and balance between family and work, and finally, the Rosenberg subscales of risk to have anxiety and depression, with nine items each and dichotomic responses [70]. This research followed the Declaration of Helsinki and the Data Protection Law 3/2018 following these codes and receiving approval (Ref. 4258), being updated in 2021 (Ref. 4950).

For this study, the qualitative variables, such as country or teachers’ perspectives regarding ICTs, were calculated via frequencies (absolute and relative) and the median. For the quantitative variables, which were the exact level obtained in Rosenberg subscales of risk to have anxiety and depression [70], the mean, standard deviation, and 95% confidence intervals (CI) were used. Additionally, the breakpoint established for the subscales were used as a positive diagnostic of risk for anxiety and depression (four or more for the anxiety and two or more for anxiety) were analyzed to determine associations [70]. The Kolmogorov–Smirnov test was applied and indicated that the data did not follow the normality (*p* < 0.001). The chi-square test, Mann–Whitney U test, Kruskal–Wallis, and Spearman’s correlation tests were used.

### 3.2. Bibliographic Search

Three simultaneous searches using the Scopus, Web of Science (WOS), and Medline via PubMed databases were performed using the PICO (Population, Intervention, Comparison, and Outcomes) structure. The bibliographic search was carried out using the Medical Subject Heading (MeSH) terms, which were previously selected in accordance with the research’s purposes (O3 and O4) (Table 2). Additionally, the keyword “teachers” or “academic” were incorporated in the search to gather more publications.

The bibliometric analysis was implemented in July 2021 using the research accordingly to each database. The Boolean operators chosen were “OR” and “AND”, and the fields used to identify the relevance were “title”, “abstract”, and “keywords”. For Scopus, the research included the use of abstract, title, and keywords ((TITLE-ABS-KEY (“mental health”) OR TITLE-ABS-KEY (“mental disorders”) OR TITLE-ABS-KEY (“Anxiety Disorders”) OR TITLE-ABS-KEY (“Mood Disorders”) OR TITLE-ABS-KEY (“Depressive Disorder”) OR TITLE-ABS-KEY (“Anxiety”) OR TITLE-ABS-KEY (“stress disorders, traumatic”)) AND (TITLE-ABS-KEY (“universities”) OR TITLE-ABS-KEY (“schools”) OR TITLE-ABS-KEY (“teaching”)) AND (TITLE-ABS-KEY (“faculty”) OR TITLE-ABS-KEY (“teachers”) OR TITLE-ABS-KEY (“academics”)) AND (TITLE-ABS-KEY (“technology”) OR TITLE-ABS-KEY (“Educational Technology”) OR TITLE-ABS-KEY (“Computer User Training”) OR TITLE-ABS-KEY (“Models, Educational”) OR TITLE-ABS-KEY (“ICTs”))). In the case of WOS, the research was implemented focusing on the topic as the descriptor obtaining the following research strategy: TS = ((“mental health” OR “mental disorders” OR “Anxiety Disorders” OR “Mood Disorders” OR “Depressive Disorder” OR “Anxiety” OR “stress disorders, traumatic”) AND (“universities” OR “schools” OR “teaching”) AND (“faculty” OR “teachers” OR “academics”) AND (“technology” OR “Educational Technology” OR “Computer User Training” OR “Models, Educational” OR “ICTs”)). Finally, the Medline research was based on the use of the MeSH terms and other terms obtaining the following strategy: ((Mental health[MeSH Terms]) OR (mental disorders[MeSH Terms]) OR (anxiety disorders[MeSH Terms])) OR (mood disorders[MeSH Terms]) OR (depressive disorder[MeSH terms]) OR (anxiety[MeSH Terms]) OR (stress disorders, traumatic[MeSH Terms]) AND (universities[MeSH Terms]) OR (schools[MeSH Terms]) OR (teaching[MeSH Terms]) AND ((faculty[MeSH Terms])) OR (teachers[Other Term]) OR (academic[Other Term]) AND ((technology[MeSH Terms]) OR (educational technology[MeSH Terms]) OR (Computer User Training[MeSH Terms]) OR (Models, Educational[MeSH Terms]) OR (ICTs[Other Term])).

The exclusion criteria were publications whose population were students, teachers from secondary or primary level, studies over 20 years, studies that did not include the technology, or whose topic concentrates on patients. Selected studies were those related to teachers from the university level’s use of ICTs and the influence of mental health.

**Figure 1:**
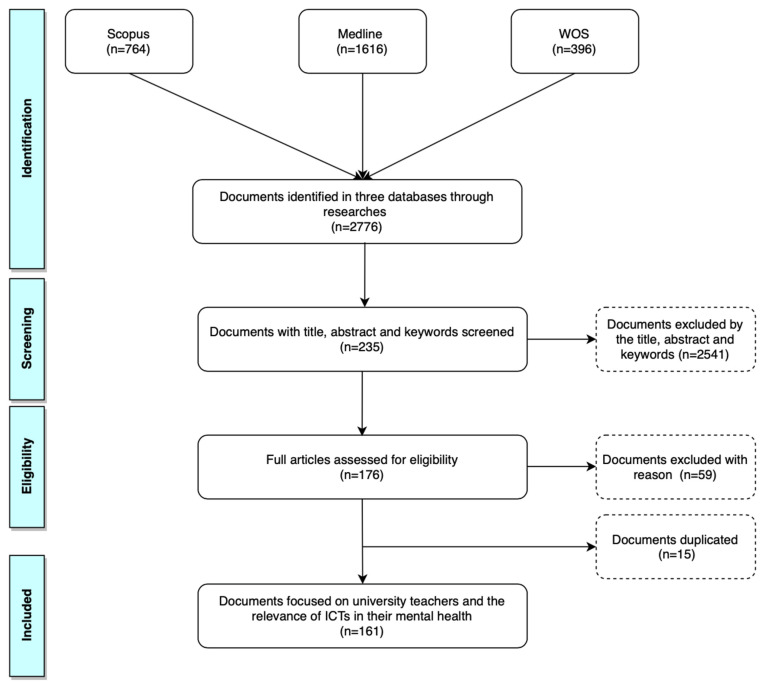
Flow diagram followed for collecting data using the stages of identification, screening, eligibility, and inclusion.

Before 1 July 2021, there were identified 2776 documents published, that addressed the topic of teachers, mental health, and technology. During the screening, 2541 studies were eliminated from the study since in their titles, abstracts, and/or keywords referred to different populations (such as teachers from high schools), intervention (that did not include any type of technology) or outcomes (related to the mental health or emotional distress). The following stage, further articles were excluded according to content of the text and timeframe, articles whose population were teachers of secondary level or the lack of inclusion of ICTs. In this phase, 59 documents were eliminated, also excluding 15 papers that were duplicated (Figure 1).

The statistic package called SPSS version 24 (IBM Corporation, Armonk, NY, USA), VOSviewer version 1.6.15 (Ness Jan van Eck, The Netherlands), and Excel version 17 (Microsoft Corporation, Redmond, Washington, USA) were implemented to study the data obtained after the screening of the search (Figure 1). Additionally, with the information provided by the databases, the Clarivate Journal Citation Report was used to define which journal was indexed, the Journal Impact Factor of the year of publication, the quartile of the journal, and the JIF percentile. Relative frequencies and medium were implemented for qualitative variables, i.e., country, journal, or year of publication. Based on the normalization test (*p* < 0.001), Mann–Whitney, chi-square U, Kruskal–Wallis tests, and Spearmen’s correlation were applied for the quantitative variables of the 161 documents (Appendix A Table A1), Additionally, the Strengthening the Reporting of Observational Studies in Epidemiology (STROBE) Checklist [71] was applied for the analysis of the quality of the methodology of the top five observational studies in the area (Appendix A Table A2).

## 4. Results

### 4.1. STEM Teachers at University Level

The observational study’s initial analysis showed that 32.7% of the participants came from Spanish high education institutions, while 67.3% worked in Ecuador; 76.4% of the university teachers taught undergraduate students, with a median of 10 years of experience (34.5% had less than ten years, and 27.3% had between 10 to 20 years of experience). The mean level of anxiety was set at 6.84 ± 2.54 (95% CI 6.15–7.24); meanwhile, the mean depression level was 4.91 ± 2.89 with a 95% IC 4.13–5.69. The analysis of the anxiety and depression levels indicated that for the country, working experience, and teaching at different levels (Table 3) showed no significant differences except for the case of the depression level, which varied per country (*p* = 0.009). The mean of the depression level was 5.5 ± 3.24 (95% CI 3.88–5.53) among Spanish teachers in contrast with the 4.6 ± 2.71 (95% CI 3.71–7.11) of Ecuadorian university teachers. Nevertheless, the correlations for the level of anxiety and depression were not linked to any of the previous variables (*p* > 0.05).

**Figure 2:**
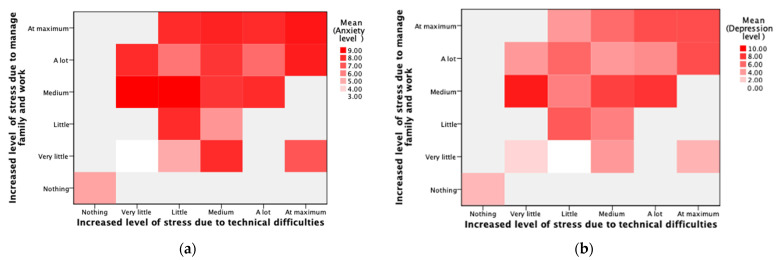
Comparison of the correlations between the technical issues and the balance between the family and work related to the anxiety (**a**) and depression levels (**b**) through a heat map.

The anxiety and depression levels of the teachers were linked to the perception regarding the ICTs, the availability of resources, lacking resources as an obstacle, and COVID-19 (*p* < 0.05) (Table 2). Additionally, COVID-19 as a stressor (presented in 78.18%) showed significance regarding the level of anxiety (*p* = 0.022) and depression (*p* = 0.006). Another factor that showed relevance for the levels of anxiety and depression was the balance between family and work (*p* = 0.01). The correlations (Table 2) indicated that the level of anxiety increased by the inability of having internet (*p* = 0.027), the lack of resources (*p* = 0.001), training (*p* < 0.001), models (*p* < 0.001), time (*p* < 0.001), research that indicates the benefits of the use of ICTs (*p* = 0.01), the stress caused by COVID-19 (ρ = 0.31; *p* = 0.021), stress related to the need to use ICTs (ρ = 0.42; *p* = 0.001), the technical issues (ρ = 0.39; *p* = 0.003), and the balance between the family and work (ρ = 0.42; *p* = 0.001) (Figure 2). Meanwhile, the depression level was linked to having less ICTs’ skills (ρ = −0.39; *p* = 0.011), the lack of resources (*p* = 0.033), training (*p* = 0.025), models (*p* = 0.004), time (*p* = 0.033) (Table 2), the stress caused by COVID-19 (ρ = 0.38; *p* = 0.005), stress related to the need to use ICTs (ρ = 0.43; *p* = 0.001), the technical issues (ρ = 0.43; *p* = 0.001), and the balance between the family and work (ρ = 0.42; *p* = 0.001) (Figure 2).

Based on the levels of anxiety and depression, the risk of developing each was analyzed. The risk of developing anxiety among the teachers was 85.5%, being presented as similar frequency among Spanish (83.3%) and Ecuadorian (86.5%) (*p* > 0.05). The risk of developing anxiety showed significant differences in the role of ICTs in education and lack of ICTs (resources, software, training, models, time, and research about the benefits of ICTs) (*p* < 0.05) (Table 4). Additionally, COVID-19 as a stressor showed a significant difference between the teachers with a risk of anxiety (82.98%) (X^2^ = 8.03; *p* = 0.005). Meanwhile, the risk of depression was related to COVID-19 as a stressor (X^2^ = 4.39; *p* = 0.037), the balance between family and work (X^2^ = 16.3; *p* = 0.006), availability of computers and internet, the role of the ICTs in education, lack of models, and time (*p* < 0.05) (Table 3).

Furthermore, the correlations were analyzed for each variable and the risk of anxiety and depression (Table 4). The anxiety risk was linked to the frequency of using virtual environments (ρ = 0.30; *p* = 0.026), the lack of resources (*p* = 0.032), training (*p* < 0.001), models (*p* < 0.001), time (*p* = 0.001), and research about the benefits of using ICTs in the education (*p* < 0.001) (Table 4). Additionally, COVID-19 (*p* = 0.037), need to use ICTs (*p* = 0.005), technical issues (*p* = 0.005), and the balance between family and work (*p* = 0.006) were linked to having a higher risk of anxiety (Table 5). Besides, risk of having depression (Table 4) was linked to the unavailability of internet (ρ = −0.67; *p* = 0.049), less ICTs’ skills (ρ = −0.34; *p* = 0.011), the lack of resources (*p* = 0.038), training (*p* = 0.025), and models (*p* = 0.006). This risk was associated with COVID-19 as a stressor (*p* = 0.004), need to use ICTs (*p* = 0.001), technical issues (*p* = 0.007), and the balance between family and work (*p* = 0.001) were linked to having a higher risk of anxiety. Finally, the risk of anxiety and depression was connected (ρ = 0.59; *p* < 0.001).

Finally, a multivariant analysis based on linear regression for positive risk of anxiety among STEM teachers at university level indicated (R^2^ = 0.62; *p* = 0.016) that this risk is dependent on lack of time (*p* < 0.001), software (*p* = 0.016), and research that clarified the benefits of using ICTs (*p* = 0.005); this risk is codependent of the chance to developing depression (*p* < 0.001).

### 4.2. Bibliographic Search

**Figure 3:**
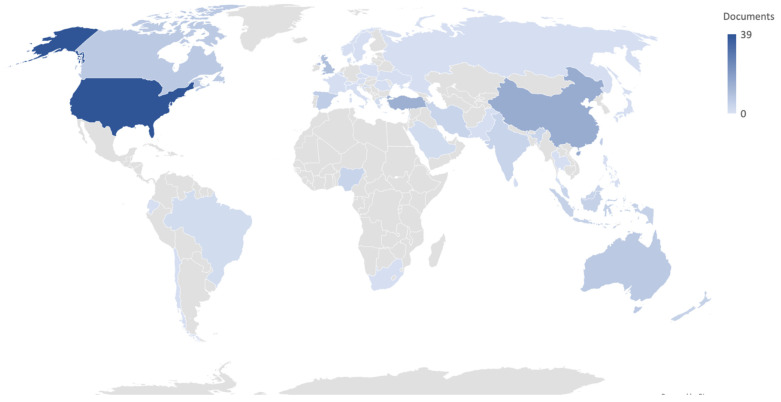
Distribution of the documents per country.

The analysis of the bibliometric method reflected that most investigations were carried out in the United States (USA, with 39 documents), followed by China (with 15 papers), Turkey (with 14 documents), the United Kingdom (10 articles), and other countries (Spain and Ecuador with less than five documents each) with few publications on the topic studied (Figure 3). This figure presents the nations from whom the authors have published the documents (*N* = 161), being more often the case of countries, such as Japan or Chile, that provided only one paper. The number of publications per year and citations showed significant differences between countries (*p* = 0.01), with the USA being the most influential producer of publications per year (*p* = 0.003), indexing most of the publications (*p* = 0.011) in higher quartiles (*p* = 0.043), and higher JCR (*p* = 0.048). Despite this, the frequency of publications in the latest years indicated that countries with fewer investigations have increased during the last three years (ρ = −0.87; *p* < 0.001).

Nevertheless, when analyzed per continent and the year, the number of citations, journal citation report (JCR), and percentile of the journal, there were no significant differences (*p* = 0.71). There were substantial differences between continents regarding whether the journal was indexed (X^2^ = 12.54; *p* = 0.014) and the quartile (X^2^ = 32.76; *p* = 0.036). The correlations indicated how other continents such as Oceania had increased the rate of publications mainly in the last years (*p* = 0.033) but had more minor citations (*p* < 0.001), indexed journals (*p* = 0.005), and published articles in lower quartiles (*p* = 0.002) than compared to other continents, such as Europe.

**Figure 4:**
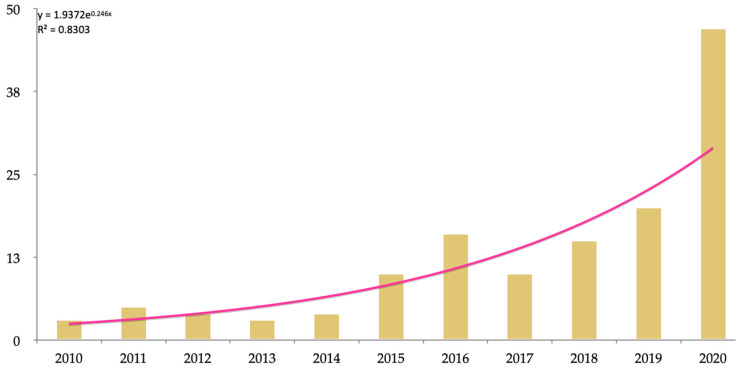
Distribution of the documents per year and exponential curve of publication.

Another aspect analyzed was the frequency of publications per year (Figure 4), whose highest rate was achieved in 2020, the year of the COVID-19 pandemic (29.6% of the publications made in the last 20 years). The analysis indicated that there was a significant difference between the year of publication and quartile of the article (*p* < 0.001) and the number of citations (*p* < 0.001). The correlations indicated that the latest publications had more minor citations (ρ = −0.47; *p* < 0.001), although the rate of publications has no significant differences (*p* = 0.01). The median of the year of publications was 2018 (*p* = 0.05); from this year, as a break point, the number of citations amplified faster (*p* = 0.032). This change (Figure 3) had similarities with the year 2015 that had 10 documents, although there was a decrease of six papers from 2016 to 2017.

The number of citations of each article diverged according to the theme (*p* < 0.001), with a mean of 8.2 citations (SD = 15.47; IC 95% = 10.63–5.77). The citations per document were linked to journal indexed, JCR of the journal of the year of publication, quartile, and percentile (*p* < 0.001). According to the citations, the top ten articles were reviews, two observational studies, two guidelines or theoretical studies, and two qualitative studies. The results indicated that this area is more common than the reviews.

Based on the citations and the type of studies, the top five observational or cross-sectional studies with a higher number of citations were analyzed (Table 6). The most cited article, with 138 citations and from Turkey, focused on determining the relationship between attitude to computers and anxiety for pre-teachers at the university level [72]. This article highlights the importance of computer self-efficacy and anxiety related to using such technology as predictors in the education that this teacher will provide. For teachers and education, this article clarifies how the attitude, including the technostress and ICTs’ skills, are intrinsic factors related to the mental distress that university teachers may experience. The second article focused on nursing teachers and how ICTs are a risk factor for technostress [73]. The third focused on university teachers, in which it was linked the use of ICTs for the different functions and ICTs’ skills, and the anxiety related to ICTs [74]. The fourth and fifth focused on how the use of ICTs in the future seems to be determined by anxiety caused by self-efficacy, behavior, or willingness [75,76]. This table displayed how more relevant observational research were published in Turkey (3/5), which is also the third country with a higher number of articles which focused on the topic, analyzed through the bibliometric analysis. Additionally, it is reflected in the top articles the existing link between an individual’s skills and perception regarding ICTs and the risk of technostress; furthermore, the fourth most cited article showed how teachers whose work is related to STEM education have lower levels of anxiety or stress related to ICTs.

Besides, the quality of the methodology was analyzed (Table 5), indicating that despite having more minor citations, the second most cited article [73] had over 70% in the quality of the methodology, followed by the first article with a higher number of citations [72]. The third article with the most methodological quality was published in 2016 and had the third-ranking number of citations [74]. The remaining papers with fewer citations, and more recently, had less than 50% of methodological quality. These results indicate that the number of citations and methodological quality are critical factors in the relevance of the research published.

**Figure 5:**
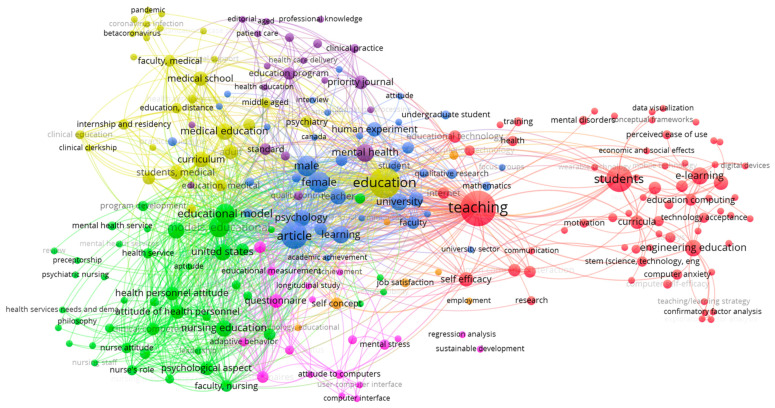
Co-occurrence of the repetitive terms identified per article.

The analysis of index keywords of each document, based on a minimum of two nodes, showed seven clusters as the most concurrency topics (Figure 5). The issues identified seven groups (Table 7) (formed by 224 keywords indicated with 4819 links and a total of 7350 ties). The first and foremost cluster (representing 30.49% and being in red) focused on educational technology, STEM education with great emphasis on students, and teachers’ self-efficacy (presenting 17.56% of co-occurrence among the keywords). This red cluster represented one of the main sub-topics based on the technology, teaching, and students focusing on STEM. The next sub-topic (green), conformed by 44 keywords (19.73%), has a central theme regarding the educational model in nursing education and the relevance of the management and organization for the psychological impact. In higher educational institutions, the third cluster (in blue), which represented 16.59% of keywords, focused on psychological issues, especially anxiety. The fourth cluster, in yellow, with the exact representation as to the third, represented the sub-topic of mental health in the health faculty, specifically the medical education, focusing on the distance education caused by the coronavirus and the psychological adaptation. The following cluster (in purple), with 20 keywords and an occurrence of 8.97%, concentrated on mental health in the health faculty and its relationship with practical education in hospitals. The sixth cluster, formed by 8.52% of the keywords and represented in pink, focused on mental stress and technology. The last cluster (orange) was created by seven keywords and presented 3.14% of weight from the seven clusters and was centered on sciences, self-concept, and the psychology in the educational sector.

## 5. Discussion

The mixed-method analysis has identified several important aspects. First, from the descriptive analysis, it can be resumed in the link between anxiety levels, depression, and various impediments for online education, such as the lack of training, and the relevance of other stressors like COVID-19 or the balance between family and working, these being results independent from the country. Meanwhile, the bibliometric analysis had highlighted the lack of studies that focused on measuring the STEM teachers at the university level of mental discomfort or problems, and instead identified the STEM training that these workers have as a protective factor. Few studies have been carried out in Spain or Ecuador focusing on this relevant topic, and much less were intercontinental studies identified.

The data from the observational analysis has highlighted how STEM teachers at the university level had a high level of anxiety and depression, with the risk of developing them as a disorder elevated. The mental well-being of Spanish and Ecuadorian teachers seemed to be related to working conditions and workers’ attitudes. These workers indicated that the impact of COVID-19, the imposition of using ICTs for online teaching, the technical problems, and the balance between the familiar and working environments were critical features for their stress.

There were no significance differences according to country or working experience for the anxiety and depression level; rather, these levels were associated with the perception of the STEM teachers at higher education institutions concerning diverse difficulties, such as lack of resources, training, or time. Moreover, the ICTs’ skills, described as moderate-high, were not linked to anxiety or depression. This result indicated that previous skills to the pandemic seemed to have not been a protective factor against mental issues. The associations previously described may reflect how the transfer from face-to-face to online teaching was a stressor independently of the previous training in STEM education or previous ICTs’ skills. These outcomes are in accordance with other researchers [77,78], whose findings showed the need for training as a vital element to prevent technostress among teachers. Furthermore, another study carried out in Ecuador specified that previous training and experience with online teaching before the pandemic was a protective factor, reducing the stress level and possibly other psychological symptoms [55]. Nevertheless, the previous studies in higher education institutions did not differentiate between STEM teachers or where they teach [55,77,78].

Another interesting finding was the impact balancing family and the working environment in the mental well-being of the participants. These results were consistent with previous studies that indicated how the inclusion of the ICTs in the living environment increases the hostility and stress between the workers and the rest of the family members [77]. However, because of the COVID-19 restrictions, the anxiety levels of these workers continue for a long time because of the obligation to balance workload combined with the work of carrying out family care duties [79]. This imposition to balance the work demands and family duties could be one of the reasons why teachers working in the home environment indicated having higher levels of emotional issues and psychological symptoms [80]. Several studies indicated diverse psychological symptoms, mainly anxiety and stress, among teachers at different educational levels and at the university [81]. In this sense, a Chinese study indicated that posttraumatic stress disorder was around 25% among college teachers, reflecting that the incidence of mental health issues is higher in this group [23].

The current observational analysis indicated that 85% of the university teachers were at risk of developing anxiety as chronic mental health, which was also connected to depression [82,83]. The relationship between anxiety and depression, established long ago [84], was also associated with lack of time, specific software, and research that clarified the benefits of using technology. This relationship between anxiety, depression, and these obstacles could be different and be more conditional depending on the previous background or new stressors such as the impact of the pandemic. However, other studies indicated that STEM teachers had less risk of anxiety related to ICTs [72], being contradictory to the current studies since the STEM teachers also had high levels of anxiety. Other articles that analyzed the relationship between technostress and COVID-19 suggested that age or gender play a role in the anxiety linked to the technology [32,67], although these studies used only technostress and the possible effect of the family/work—the stress caused by COVID-19 or the lack of training was missing. This lack of association from previous studies has been magnified by the observational studies found in the bibliometric analysis [72,73,74,75,76]. The top five articles (Table 5) indicated that pre-service and in-service STEM teachers in higher education institutions had fewer mental issues and less risk. These articles [72,73,74,75,76] were in contract with the findings from the current observational analysis, whose findings indicated that STEM education or experience in STEM courses was not a definitive shielding factor against mental issues. In fact, the STEM teachers also suffered from psychological problems during the pandemic, which could be associated with the lack of previous experience with online learning [53].

Another aspect identified in the bibliographic analysis was the lack of studies carried out in the population selected (Spain or Ecuador), and the papers did not consider the lack of technical support as a stressor [72,73,74,75,76]. Additionally, another result was that the current investigations are more commonly reviews [85,86], being also more cited. The top publications focused on recommendations [87] to prevent mental issues, qualitative approaches, and little provided current data; being less common, the perspective of STEM teachers at university level. Other prior works indicated that the highest prevalence of work-related technostress among teachers (around 10%) was related to a specific age group (women from 30 to 39 years old with children), which could be related to training, working workload, and family chores, and the field in which the education is provided [73,87]. Thus, most works focused on analyzing prevalence and its relationship with the teachers’ attitudes but did not include the teachers’ perspective regarding obstacles such as the lack of ICTs [72,73,74,75,76].

Additionally, these articles (Table 5) focused on Turkey and pre-service faculty members, not including the STEM teachers as an independent group but studying this population in combination with other educators [72,73,74,75,76]. Additionally, one study focused on healthcare teachers [73] showed that lectures from health sciences despite being close to STEM education, presents high levels of stress and anxiety linked to ICTs. These differences among teachers from higher education institutions [72,73,74,75,76] also amplifies the fact, highlighted in the observational analysis, that training with ICTs is a major factor to protect these workers [88]. In this sense, UNESCO described the relevant preventative factors and how to decrease such prevalence, and highlighted that workers need training, updating the ICTs available, and supporting the continuity of adequate education [88].

Moreover, the bibliometric analysis showed that the quality of the observational studies is under 70% despite being indexed, underlining the need to further descriptive studies with higher methodological quality. Another aspect identified in the bibliometric analysis was the fact that most of observational studies focused on STEM teachers at higher institutions previous to the pandemic, and therefore, further analysis during the current pandemic are needed. In this sense, the current observational analysis might provide more relevant data, although such information and its application in the education field (such as increasing the training with ICTs and its later analysis via experimental study) should be made with precautions based on the reduced number of participants.

Despite the findings and application to the field, the current research also has limitations. The major limitation of this study is its small sample of teachers, although no previous research has focused only on STEM teachers during COVID-19 at the university level. This limitation is associated with the population and the rate of response from participants, being under 10%. Moreover, another limitation from the methodology is the use of a survey based on perception and the cross-sectional design that limits the results to a timeframe. Besides, the data from university teachers has limited the results to the countries that participated, so the extrapolation of the data needs to be carefully carried out. Additionally, some personal data of the sample were not included, such as gender, number of children or their age, which could provide further information. Additionally, since the research method was based on a mixed approach, another weakness of the methodology could be the choice of MeSH or other keywords not being included, which might have enclosed the number of publications and therefore, the possibility of framing the survey results into the current knowledge.

Nonetheless, the current research based on a mixed-method has raised interesting questions about mental health and its relationship with the ICTs, taken in educational higher institutions environment during the pandemic, and e-learning education. Despite these limitations and based on the topic, timeframe, population, and sample size, including a few publications, the findings presented with the mixed-method could provide further knowledge in this area. The results, mainly through the bibliographic analysis, highlight that previous studies were not focusing on STEM teachers and there was a lack of data in this specific population, as well as stressors that also affect these workers. A vital practical repercussion is the essential nature of training and adequate technical support as well as giving mental support to the workers in the educational sector.

## 6. Conclusions

Among university teachers, the anxiety and depression levels and their risk of developing them as a mental disorder from a sample of STEM Spanish and Ecuadorian teachers at university were highly elevated and linked to their perspective. One of the most relevant findings is that anxiety and depression can be linked to various obstacles, such as lack of training, resources, time, or research. The results showed that STEM teachers at university level perceived COVID-19, the imposition of using ICTs, the technological issues, the balance between family and work, and their perception of the lack of means as obstacles towards the mental issues among these workers. These results are fascinating since the teachers, independently of the country, indicated that their mental health and stress were high, caused by different factors related to technology. Other factors were also noteworthy, such as the impact of COVID-19, since these data indicated their relevance on mental health, although these factors were not conclusive in the risk of anxiety and depression. The second significant finding was that the described variables (e.g., training, or behavioral attitudes regarding ICTs) in most significant previous works were connected to a higher risk of stress and anxiety associated with technology.

Another important finding that emerged from the bibliometric analysis was that most results focused on countries such as Turkey or the USA, with a lack of research carried out in Spain or Ecuador. Moreover, there was also a lack of observational analysis whose focus was determining factors linked to mental distress and risk factors. Most of the documents were reviews that analyzed the impact of ICTs and stress, and highlighted how STEM teachers have lower risk of mental issues, which could be not applicable in the current pandemic. These results of the previous observational studies, independently of the year or number of citations, in contrast with the observational data from the current research, did not analyze the effect of STEM education as a preventive factor related to the training of teachers. Finally, all these findings suggest that to decrease the prevalence of mental issues, greater compliance with the relevant measures, mainly training with ICTs, is needed, along with further research that focuses on whether such actions are implemented.

## Figures and Tables

**Table 1:** Initial data of the procedure and the study population.

Procedure	*N*	Responses	Completed the Surveys	Frequencies of Response
Sent out survey email invitation to STEM departments (i.e., engineering or physics) from the Engineering School	24 email survey invitations sent, which were resent by the teachers to other colleges	Three centers for Spain (Valencia, Seville, and Cordoba) One center for Ecuador	-	60% of Spanish
				100% of Ecuadorian centers
Average response of the surveys	173 teachers accessed the surveys	45 in Spain 45 in Ecuadorian center	18/45 completed the survey in Spain 37/45 completed the survey in Ecuador	40% in Spain 82.2% in Ecuador
**Variables**		**Teachers that Completed the Survey (*N* = 55)**		
		***N***	**Frequencies**	
Working experience		19 with less than 10 years 15 with an experience between 10 to 20 11 with an experience between 20 to 30 10 with more than 30 years	34.5% with less than 10 years 27.3% with an experience between 10 to 20 20.0% with an experience between 20 to 30 18.2% with more than 30 years	
Teaching level		42 undergraduate 13 postgraduate	76.4% undergraduate 23.6% postgraduate	
ICTs’ skills		11 indicted enough 32 indicated numerous 12 indicated outstanding	20% indicted enough 58.2% indicated numerous 21.8% indicated outstanding	

Note: The estimation of the sample was based on previous intercontinental researchers [59], with 6% of anxiety related to new information and communication technologies (ICTs) [67] and having an expected rate of response set at 5% per center [68].

**Table 2:** Description of MeSH terms.

Medical Subject Heading (MeSH) Terms	Description
Mental Health	“Emotional, psychological, and social well-being of an individual or group”
Mental Disorders	“Psychiatric illness or diseases manifested by breakdowns in the adaptational process expressed primarily as abnormalities of thought, feeling, and behavior producing either distress or impairment of function”
Anxiety Disorders	“Persistent and disabling anxiety”
Mood Disorders	“Those disorders that have a disturbance in mood as their predominant feature”
Depressive Disorder	“An affective disorder manifested by either a dysphoric mood or loss of interest or pleasure in usual activities. The mood disturbance is prominent and relatively persistent”
Anxiety	“Feelings or emotions of dread, apprehension, and impending disaster but not disabling as with anxiety disorders”
Stress Disorders, Traumatic	“Anxiety disorders manifested by the development of characteristic symptoms following a psychologically traumatic event that is outside the normal range of usual human experience. Symptoms include re-experiencing the traumatic event, increased arousal, and numbing of responsiveness to or reduced involvement with the external world. Traumatic stress disorders can be further classified by the time of onset and the duration of these symptoms”
Universities	“Educational institutions providing facilities for teaching and research and authorized to grant academic degrees”
Schools	“Educational institutions”
Teaching	“A formal and organized process of transmitting knowledge to a person or group”
Faculty	“Teaching and administrative staff having academic rank in a post-secondary educational institution”
Technology	“The application of scientific knowledge to practical purposes in any field. It includes methods, techniques, and instrumentation”
Educational Technology	“Systematic identification, development, organization, or utilization of educational resources and the management of these processes. It is occasionally used also in a more limited sense to describe the use of equipment-oriented techniques or audiovisual aids in educational settings”
Computer User Training	“Process of teaching a person to interact and communicate with a computer”
Models, Educational	“Theoretical models which propose methods of learning or teaching as a basis or adjunct to changes in attitude or behavior. These educational interventions are usually applied in the fields of health and patient education but are not restricted to patient care”

**Table 3:** Differences and correlations of the variables and the anxiety and depression levels.

Variables	Anxiety Level		Depression Level	
	Differences	Correlation (*p*-Value)	Differences	Correlation (*p*-Value)
Country	0.38	−0.70 (0.61)	0.009	−0.17 (0.21)
Working experience	0.17	−0.12 (0.93)	0.59	−0.043 (0.76)
Teaching at different levels	0.77	0.59 (0.66)	0.41	−0.15 (0.41)
Role of the ICTs in the education	0.67	0.45 (0.001)	0.045	0.29 (0.033)
Availability of computer	0.016	−0.07 (0.63)	0.027	0.028 (0.84)
Availability of internet	0.031	−0.29 (0.027)	0.45	−0.23 (0.096)
Frequency of using ICTs (virtual environments)	0.037	0.16 (0.25)	0.11	−0.26 (0.053)
Lack of resources	0.013	0.48 (0.001)	0.08	0.29 (0.033)
Lack of software	0.012	0.37 (0.005)	0.039	0.25 (0.071)
Lack of training	0.003	0.49 (<0.001)	0.43	0.31 (0.025)
Lack of models	<0.001	0.55 (<0.001)	0.025	0.38 (0.004)
Lack of time	0.002	0.49 (<0.001)	0.022	0.29 (0.033)
Lack of evidence	0.01	0.35 (0.01)	0.021	0.25 (0.071)

**Table 4:** Variables that indicated significant differences for the risk of anxiety and depression.

Variables	Answers	Risk of Anxiety		*p*-Value	Risk of Depression		*p*-Value
		Yes	No		Yes	No	
Availability of computer	Rather not say	0 (0%)	0 (0%)	0.081	0 (0%)	0 (0%)	0.004
	Nothing	2 (4.26%)	2 (25.00%)		0 (0.0%)	4 (22.22%)	
	Little	5 (10.64%)	0 (0.0%)		5 (13.51%)	0 (0.0%)	
	Enough	10 (21.28%)	0 (0.0%)		9 (24.32%)	1 (5.56%)	
	A lot	30 (63.83%)	6 (75.00%)		23 (62.16%)	6 (72.22%)	
Availability of internet	Rather not say	0 (0.0%)	0 (0.0%)	0.24	0 (0.0%)	0 (0.0%)	0.048
	Nothing	0 (0.0%)	0 (0.0%)		0 (0.0%)	0 (0.0%)	
	Little	0 (0.0%)	0 (0.0%)		0 (0.0%)	0 (0.0%)	
	Enough	8 (15%)	0 (0.0%)		7 (18.92%)	0 (0.0%)	
	A lot	40 (85%)	8 (100%)		30 (81.08%)	8 (100%)	
Role of the ICTs in the education	No	41 (87.23%)	6 (75.00%)	0.002	31 (83.78%)	16 (88.89%)	0.029
	Maybe	0 (0.0%)	2 (25.00%)		0 (0.0%)	2 (11.11%)	
	Yes	6 (12.77%)	0 (0.0%)		6 (16.22%)	0 (0.0%)	
Lack of resources	No obstacle	5 (10.64%)	4 (50.00%)	0.042	4 (10.81%)	5 (27.78%)	0.152
	Less important	2 (4.26%)	0 (0.0%)		2 (5.41%)	0 (0.0%)	
	Important in some cases	12 (25.53%)	1 (12.50%)		7 (18.92%)	6 (33.33%)	
	Considerably important	6 (12.77%)	2 (25.00%)		5 (13.51%)	3 (16.67%)	
	Highly important	22 (46.81%)	1 (12.50%)		19 (51.35%)	4 (22.22%)	
Lack of software	No obstacle	2 (4.26%)	4 (50.0%)	0.003	1 (2.70%)	5 (27.78%)	0.056
	Less important	3 (6.38%)	0 (0.0%)		2 (5.41%)	1 (5.56%)	
	Important in some cases	13 (27.66%)	0 (0.0%)		10 (27.03%)	3 (16.67%)	
	Considerably important	11 (23.40%)	2 (25.0%)		8 (21.62%)	5 (27.78%)	
	Highly important	1838.30%)	2 (25.0%)		16 (43.24%)	4 (22.22%)	
Lack of training	No obstacle	1 (2.1%)	4 (50.0%)	<0.0001	1 (2.70%)	4 (22.22%	0.1
	Less important	3 (6.4%)	1 (12.5%)		3 (8.11%)	1 (5.56%)	
	Important in some cases	12 (25.5%)	2 (25.0%)		8 (21.62%)	6 (33.33%)	
	Considerably important	10 (21.3%)	1 (12.5%)		8 (21.62%)	3 (16.67%)	
	Highly important	21 (44.7%)	0 (0.0%)		17 (45.95%)	4 (22.22%)	
Lack of models	No obstacle	1 (2.13%)	4 (50.00%)	<0.0001	1 (2.70%)	4 (22.22%)	0.021
	Less important	2 (4.26%)	0 (0.0%)		2 (5.41%)	0 (0.0%)	
	Important in some cases	10 (21.28%)	3 (37.50%)		7 (18.92%)	6 (33.33%)	
	Considerably important	15 (31.91%)	1 (12.50%)		10 (27.03%)	6 (33.33%)	
	Highly important	19 (40.43%)	0 (0.0%)		17 (45.95%)	2 (11.11%)	
Lack of time	No obstacle	1 (2.13%)	4 (50.00%)	<0.001	1 (2.70%)	4 (22.22%)	0.065
	Less important	1 (2.13%)	2 (25.00%)		1 (2.70%)	2 (11.11%)	
	Important in some cases	9 (19.15%)	0 (0.0%)		7 (18.92%)	2 (11.11%)	
	Considerably important	16 (34.04%)	1 (12.50%)		14 (37.84%)	3 (16.67%)	
	Highly important	20 (42.55%)	1 (12.50%)		14 (37.84%)	7 (38.89%)	
Lack of evidence	No obstacle	3 (6.38%)	5 (62.50%)	0.001	2 (5.41%)	6 (75.00%)	0.1
	Less important	3 (6.38%)	1 (12.50%)		3 (8.11%)	1 (12.50%)	
	Important in some cases	17 (36.17%)	2 (25.00%)		14 (37.84%)	5 (62.50%)	
	Considerably important	12 (25.53%)	0 (0.0%)		9 (24.32%)	3 (37.50%)	
	Highly important	12 (25.53%)	0 (0.0%)		9 (24.32%)	3 (37.50%)	

**Table 5:** Variables that indicated significant differences for the anxiety and depression levels.

Variables	Risk of Anxiety		Risk of Depression	
	Correlation	*p*-Value	Correlation	*p*-Value
Lack of resources	0.029	0.032	0.28	0.038
Lack of software	0.21	0.13	0.26	0.053
Lack of training	0.48	<0.001	0.30	0.001
Lack of models	0.49	<0.001	0.37	0.006
Lack of time	0.43	0.001	0.15	0.27
Lack of evidence	0.49	<0.001	0.24	0.076
COVID-19 as a stressor	0.28	0.037	0.38	0.004
Imposition of ICTs as a stressor	0.37	0.005	0.43	0.001
Technical difficulties as a stressor	0.37	0.005	0.36	0.007
Balance between family and work as a stressor	0.36	0.006	0.43	0.001

**Table 6:** The five most-cited observational articles of the bibliometric analysis.

Title	Year	Country	Sample	Variables	Results	Source	Citations	STROBE ^1^ Checklist
Attitudes to technology, perceived computer self-efficacy, and computer anxiety as predictors of computer-supported education [72]	2012	Turkey	Pre-service teachers at the university level (*N* = 471)	Sociodemographic data, studies, department, Technology Attitude Scale, Perceived Computer Self-Efficacy Scale, Computer Anxiety Scale, and The Attitude Scale toward Applying Computer Supported Education	A model created indicated the effect level of the latent variables of attitudes to technology, computer anxiety, perceived computer self-efficacy, and the attitude toward computer-supported education on each other and their ratios.	Computers & Education	138	20/32 (62.5%)
The incidence of technological stress among baccalaureate nurse educators using technology during course preparation and delivery [73]	2005	United States	Full-time nurse educators (*N* = 115)	Nurse educator technostress scale (NETS) and demographic characteristics	The use of technology in the classroom was a significant predictor of nurse educators’ technological stress.	Journal of Nursing Education	31	25/33 (75.76%)
A study on academic staff personality and technology acceptance: The case of communication and collaboration applications [74]	2019	Romania	University teachers (*N* = 1816)	The use of the online communication and collaboration applications scale, The Unified Theory of Acceptance and Use of Technology Scale, Technology anxiety scale, and The Utrecht Work Engagement Scale	ICTs for teaching and researching depend on technology anxiety and self-efficacy.	Computers & Education	22	15/32 (46.87%)
Influential factors on pre-service teachers’ intentions to use ICT in future lessons [75]	2016	Turkey	Pre-teachers at different educational levels and university teachers (*N* = 2904)	Preservice Teachers ICT Acceptance Scale was used and included: perceived usefulness, ease-of-use, and efficacy, social influence, facilitating conditions, and computer anxiety	There was an inverse correlation between anxiety and ICT integration. There was also a negative relationship between anxiety and the teachers from scientific departments or STEM backgrounds.	Computers in Human Behavior	18	19/33 (59.59%)
A model for pre-service teachers’ intentions to use ICT in future lessons [76]	2017	Turkey	Pre-service university teachers (*N* = 199)	A design scale that included ICTs perceive usefulness, perceived ease-of-use, social influence, facilitating conditions, computer self-efficacy, attitude towards computers, anxiety, and behavioral intention	The intention of using ICTs seems to be regulated by perceived usefulness, computer self-efficacy attitude towards computers, anxiety, and behavioral intention	Interactive Learning Environments	15	13/32 (40.63%)

^1^ Strengthening the Reporting of Observational Studies in Epidemiology: STROBE Checklist.

**Table 7:** Main keywords used by the communities detected in the topic.

Cluster	Color	Weight (%)	Connection between Clusters (Links per Keyword inside Each Cluster)	Main Keywords	Topic
1	Red	30.49	1638 (17.56%)	Education technology-information technology-research-STEM- students-self-efficacy	Technology in education
2	Green	19.73	2123 (22.76%)	Educational model-health personnel attitude-nursing education-organization and management-psychological aspect	Educational model in nursing and the management for the psychological impact
3	Blue	16.59	1504 (16.12%)	Faculty-learning-university- teacher-psychology-anxiety	Mental disorders in higher education institutions
4	Yellow	13.00	2171 (23.27%)	Adaptation, psychological-education-medical school-coronavirus	Distance education caused by the coronavirus and the psychological transformation in medical education
5	Purple	8.97	888 (9.52%)	Health education-educational program-mental health-university hospital-clinical practice	Education in health field, especially for clinical practices, and impact on mental health
6	Pink	8.52	740 (7.93%)	Adaptative behavior-mental stress-stress-psychological-computer assisted instruction	Mental health and technology
7	Orange	3.14	263 (2.82%)	Psychology, education-science-self-concept-job satisfaction	Sciences, self-concept, and psychology in the educational sector

## Data Availability

The data are available; please contact the authors.

## Appendix A

**Table A1:** Correlation and *p*-values between the variables of the bibliometric analysis.

Variables	Year	Number of Citations	Indexed	JCR of the Year of Publication	Quartile	Percentile	Continent	USA vs. Other Countries	The Year of the Pandemic	Median of the Year of Publication (2018)
Year	−	−	−	−	−	−	−	−	−	−
Number of citations	−0.39 (*p* < 0.001)	−	−	−	−	−	−	−	−	−
Indexed	−0.034 (*p* = 0.67)	0.5 (*p* < 0.001)	−	−	−	−	−	−	−	−
Journal of Citation Report of the year of publication	0.16 (*p* = 0.045)	0.38 (*p* < 0.001)	0.84 (*p* < 0.001)	−	−	−	−	−	−	−
Quartile	−0.083 (*p* = 0.3)	−0.42 (*p* < 0.001)	−0.84 (*p* < 0.001)	−0.98 (*p* < 0.001)	−	−	−	−	−	−
Percentile	0.084 (*p* = 0.29)	0.42 (*p* < 0.001)	0.84 (*p* < 0.001)	0.99 (*p* < 0.001)	−0.99 (*p* < 0.001)	−	−	−	−	−
Continent	0.12 (*p* = 0.12)	−0.16 (*p* = 0.043)	−0.21 (*p* = 0.009)	−0.24 (*p* = 0.002)	0.24 (*p* = 0.002)	0.25 (*p* = 0.002)	−	−	−	−
USA vs. other countries	−0.23 (*p* = 0.003)	0.15 (*p* = 0.06)	0.20 (*p* = 0.011)	0.16 (*p* = 0.048)	−0.18 (*p* = 0.025)	−0.18 (*p* = 0.023)	0.7 (*p* < 0.001)	−	−	−
The year of the pandemic	0.87 (*p* < 0.001)	−0.39 (*p* < 0.001)	−0.027 (*p* = 0.73)	0.14 (*p* = 0.085)	−0.07 (*p* = 0.37)	0.07 (*p* = 0.4)	−0.17 (*p* = 0.037)	0.22 (*p* = 0.006)	−	−
Median of the year of publication (2018)	0.88 (*p* < 0.001)	−0.43 (*p* < 0.001)	−0.08 (*p* = 0.33)	0.08(*p* = 0.32)	−0.01 (*p* = 0.9)	−0.009 (*p* = 0.9)	0.17 (*p* = 0.031)	−0.21 (*p* = 0.008)	0.89 (*p* < 0.001)	−

**Table A2:** STROBE Checklist of the top five observational studies per item.

**Study**	**1a**	1b	**2**	**3**	**4**	**5**	**6a**	**6b**	**7**	**8a**	**8b**	**9**	**10**	**11a**	**11b**	**12a**	**12b**	**12c**
[72]	No	Yes	Yes	Yes	Yes	Yes	Yes	NP	Yes	Yes	NP	No	No	Yes	NP	Yes	Yes	No
[73]	Yes	Yes	Yes	Yes	Yes	Yes	Yes	NP	No	Yes	Yes	Yes	Yes	Yes	NP	Yes	No	No
[74]	No	Yes	Yes	Yes	Yes	No	No	NP	No	Yes	NP	No	No	No	NP	No	No	No
[75]	No	No	Yes	Yes	Yes	No	Yes	NP	No	Yes	Yes	No	Yes	Yes	Yes	No	No	Yes
[76]	No	No	Yes	Yes	Yes	No	No	NP	No	Yes	NP	No	Yes	Yes	NP	No	No	Yes
**Study**	**12d**	**12e**	**13a**	**13b**	**13c**	**14a**	**14b**	**14c**	**15**	**16a**	**16b**	**16c**	**17**	**18**	**19**	**20**	**21**	**22**
[72]	No	Yes	No	No	No	No	No	NP	Yes	Yes	No	No	Yes	Yes	No	Yes	Yes	No
[73]	Yes	No	Yes	Yes	No	Yes	Yes	NP	Yes	No	No	Yes	Yes	Yes	Yes	Yes	Yes	No
[74]	No	Yes	Yes	Yes	No	Yes	No	NP	Yes	No	No	No	Yes	Yes	Yes	Yes	Yes	No
[75]	Yes	Yes	Yes	No	No	Yes	Yes	NP	Yes	No	No	No	Yes	Yes	Yes	No	Yes	No
[76]	No	Yes	No	No	No	No	No	NP	Yes	Yes	No	No	No	Yes	Yes	No	Yes	No

Note: Not applicable = NP; Strengthening the Reporting of Observational Studies in Epidemiology = STROBE Checklist.
